# Autosomal Dominant STAT6 Gain of Function Causes Severe Atopy Associated with Lymphoma

**DOI:** 10.1007/s10875-023-01530-7

**Published:** 2023-06-14

**Authors:** Ekaterina Minskaia, Jesmeen Maimaris, Persephone Jenkins, Adriana S. Albuquerque, Ying Hong, Despina Eleftheriou, Kimberly C. Gilmour, Richard Grace, Fernando Moreira, Bodo Grimbacher, NIHR Bioresource-Rare Diseases Consortium, Zoe Adhya, Hana Alachkar, Ariharan Anantharachagan, Richard Antrobus, Gururaj Arumugakani, Chiara Bacchelli, Helen Baxendale, Claire Bethune, Shahnaz Bibi, Barbara Boardman, Claire Booth, Michael Browning, Mary Brownlie, Siobhan Burns, Anita Chandra, Hayley Clifford, Nichola Cooper, Sophie Davies, John Dempster, Lisa Devlin, Rainer Doffinger, Elizabeth Drewe, David Edgar, William Egner, Tariq El-Shanawany, Bobby Gaspar, Rohit Ghurye, Kimberley Gilmour, Sarah Goddard, Pavel Gordins, Sofia Grigoriadou, Scott Hackett, Rosie Hague, Lorraine Harper, Grant Hayman, Archana Herwadkar, Stephen Hughes, Aarnoud Huissoon, Stephen Jolles, Julie Jones, Peter Kelleher, Nigel Klein, Taco Kuijpers, Dinakantha Kumararatne, James Laffan, Hana Lango Allen, Sara Lear, Hilary Longhurst, Lorena Lorenzo, Jesmeen Maimaris, Ania Manson, Elizabeth McDermott, Hazel Millar, Anoop Mistry, Valerie Morrisson, Sai Murng, Iman Nasir, Sergey Nejentsev, Sadia Noorani, Eric Oksenhendler, Mark Ponsford, Waseem Qasim, Ellen Quinn, Isabella Quinti, Alex Richter, Crina Samarghitean, Ravishankar Sargur, Sinisa Savic, Suranjith Seneviratne, Carrock Sewall, Fiona Shackley, Ilenia Simeoni, Kenneth G. C. Smith, Emily Staples, Hans Stauss, Cathal Steele, James Thaventhiran, Moira Thomas, Adrian Thrasher, Steve Welch, Lisa Willcocks, Sarita Workman, Austen Worth, Nigel Yeatman, Patrick Yong, Sofie Ashford, John Bradley, Debra Fletcher, Tracey Hammerton, Roger James, Nathalie Kingston, Willem Ouwehand, Christopher Penkett, F. Lucy Raymond, Kathleen Stirrups, Marijke Veltman, Tim Young, Matthew Brown, Naomi Clements-Brod, John Davis, Eleanor Dewhurst, Marie Erwood, Amy Frary, Rachel Linger, Jennifer Martin, Sofia Papadia, Karola Rehnstrom, William Astle, Antony Attwood, Marta Bleda, Keren Carss, Louise Daugherty, Sri Deevi, Stefan Graf, Daniel Greene, Csaba Halmagyi, Matthias Haimel, Fengyuan Hu, Vera Matser, Stuart Meacham, Karyn Megy, Olga Shamardina, Catherine Titterton, Salih Tuna, Ernest Turro, Ping Yu, Julie von Ziegenweldt, Abigail Furnell, Rutendo Mapeta, Simon Staines, Jonathan Stephens, Deborah Whitehorn, Paula Rayner-Matthews, Christopher Watt, Emma C. Morris, Siobhan O. Burns

**Affiliations:** 1 University College London Institute of Immunity and Transplantation, London, UK; 2 Department of Immunology, Royal Free London NHS Foundation Trust, London, UK; 3 Inflammation and Rheumatology Section, University College London Institute of Child Health, London, UK; 4 Rheumatology Department, Great Ormond Street Hospital National Health Service (NHS) Foundation Trust, London, UK; 5 Clinical Immunology Laboratory, Great Ormond Street Hospital of Children NHS Foundation Trust and NIHR Great Ormond Street Hospital Biomedical Research Centre, London, UK; 6 Department of Haematology, East Sussex Healthcare NHS Trust, Saint Leonards-on-sea, UK; 7 Institute for Immunodeficiency, Center for Chronic Immunodeficiency, Medical Center, Faculty of Medicine, University of Freiburg, Freiburg, Germany

**Keywords:** Atopy, Gain-of-function, Lymphoma, STAT6

## Abstract

The transcription factor STAT6 (Signal Transducer and Activator of Transcription 6) is a key regulator of Th2 (T-helper 2) mediated allergic inflammation via the IL-4 (interleukin-4) JAK (Janus kinase)/STAT signalling pathway. We identified a novel heterozygous germline mutation *STAT6* c.1255G > C, p.D419H leading to overactivity of IL-4 JAK/STAT signalling pathway, in a kindred affected by early-onset atopic dermatitis, food allergy, eosinophilic asthma, anaphylaxis and follicular lymphoma. STAT6 D419H expression and functional activity were compared with wild type STAT6 in transduced HEK293T cells and to healthy control primary skin fibroblasts and peripheral blood mononuclear cells (PBMC). We observed consistently higher STAT6 levels at baseline and higher STAT6 and phosphorylated STAT6 following IL-4 stimulation in D419H cell lines and primary cells compared to wild type controls. The pSTAT6/STAT6 ratios were unchanged between D419H and control cells suggesting that elevated pSTAT6 levels resulted from higher total basal STAT6 expression. The selective JAK1/JAK2 inhibitor ruxolitinib reduced pSTAT6 levels in D419H HEK293T cells and patient PBMC. Nuclear staining demonstrated increased STAT6 in patient fibroblasts at baseline and both STAT6 and pSTAT6 after IL-4 stimulation. We also observed higher transcriptional upregulation of downstream genes (*XBP1* and *EPAS1*) in patient PBMC. Our study confirms STAT6 gain of function (GOF) as a novel monogenetic cause of early onset atopic disease. The clinical association of lymphoma in our kindred, along with previous data linking somatic STAT6 D419H mutations to follicular lymphoma suggest that patients with STAT6 GOF disease may be at higher risk of lymphomagenesis.

245 words.

**Supplementary Information:**

The online version contains supplementary material available at 10.1007/s10875-023-01530-7.

## Background

Atopic diseases describe conditions such as eczema, asthma, allergic rhinitis, food, drug allergy and anaphylaxis which often coexist and have shared molecular aetiology. Much of the immunological basis of atopic disease depends on interkeukin-4 (IL-4) signalling, an important cytokine in the differentiation of naive T-cells to type 2 helper T-cells (Th2) cells. IL-4 in turn is produced by Th2 cells along with other Th2 cytokines including IL-5, IL-9 and IL-13 to effect a wide range of outcomes in multiple cell types [1]. Th2 effector responses mediate immunity against extracellular parasites but also underlie many of the features associated with atopy including B cell proliferation and class-switching to immunoglobulin E (IgE), elevated numbers of eosinophils and mast cells, goblet cell hyperplasia and smooth muscle contraction [2].

**Fig. 1:**
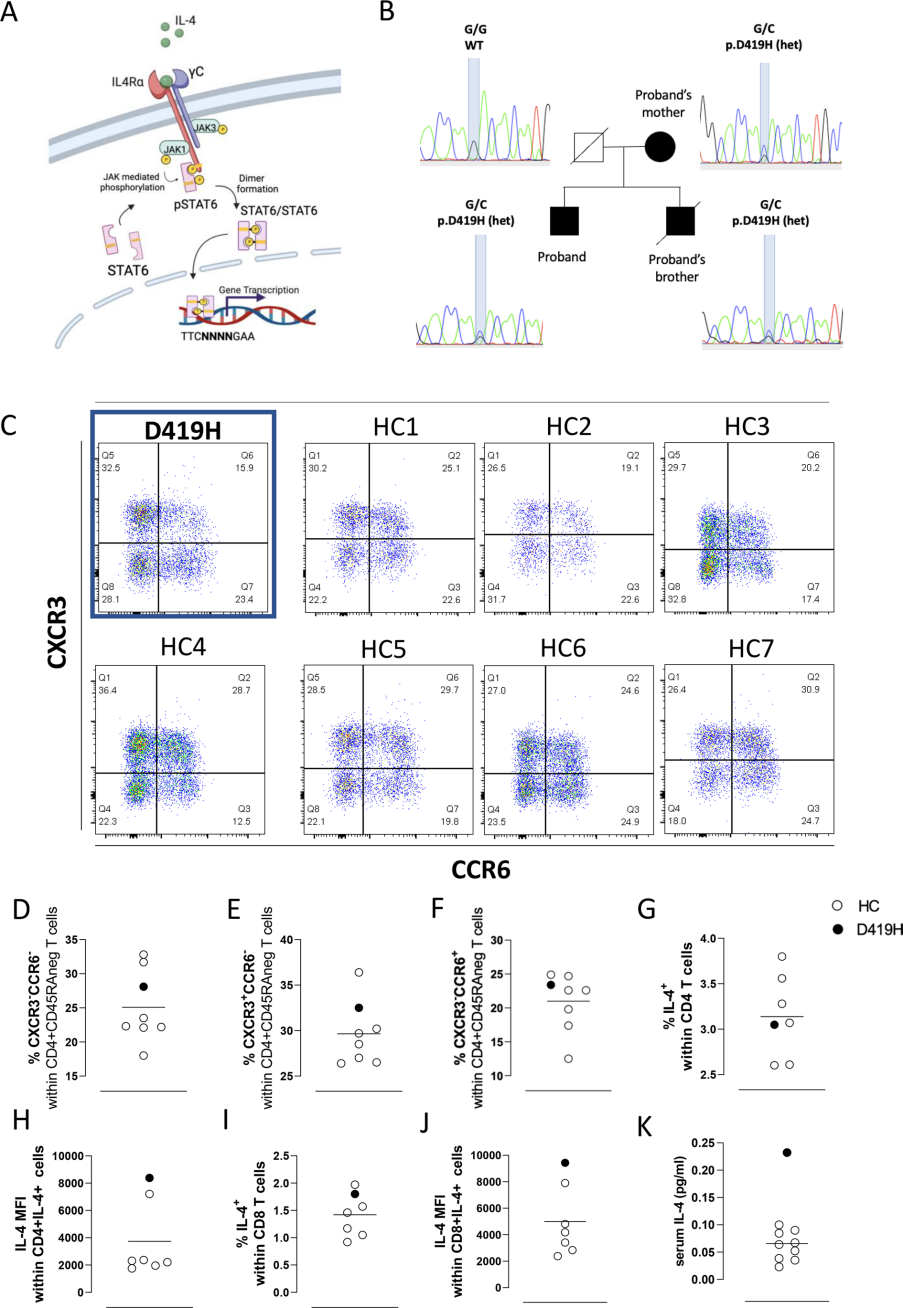
STAT6 pathway, pedigree genotyping and proband immunophenotyping. **A:** Schematic diagram of IL-4 mediated STAT6 pathway. **B:** Kindred pedigree and Sanger sequencing confirming heterozygous STAT6 D419H in affected family members. **C**: T helper (Th) subsets according to the differential expression of the chemokine receptors CCR6 and CXCR3. Frequency of CXCR3-CCR6- (**D**), CXCR3 + CCR6- (**E**), and CXCR3-CCR6 + (**F**), respectively, within CD4 memory T cells defined as CD45RA-, in the proband as compared with a cohort of healthy individuals. Flow cytometry analysis of: frequency of CD4 + T cells producing IL-4 (**G**) and mean intensity of fluorescence (MFI) within CD4 + IL4 + (**H**); frequency of CD8 + T cells producing IL-4 **(I)** and MFI within CD8 + IL4 + (**J**) cells. Serum IL-4 (**K**) as determined by multiplex electrochemiluminescence-based cytokine assay. Each dot represents one individual

IL-4 drives intracellular signalling through the janus kinase (JAK)- signal transducer and activator of transcription (STAT) pathway (Fig. 1A). Upon IL-4 receptor binding, JAK1 and JAK3 are activated and phosphorylate STAT6 on amino acid Y641 [3]. This results in homo-dimerisation and translocation of phosphorylated STAT6 (pSTAT6) into the nucleus, directing downstream differential gene expression to effect cell responses. STAT6 dimers recognise an 8–10 base-pair palindromic DNA element with a consensus sequence of 5’-TTC(NNN)GAA—3’, N indicating a spacer sequence in the element between the consensus [4]. DNA binding allows for expression of target genes, including *EPAS1*, *XBP1* and *BCL6* required for IL-4 mediated responses including Th2 cell differentiation and proliferation [5–7].

The essential role for STAT6 in transducing IL-4 signalling is highlighted by loss of function studies where STAT6 deficiency in mice impairs IL-4 driven lymphocyte proliferation, Th2 differentiation and B-cell class switching to IgE [8, 9]. Gain of lymphocyte- restricted STAT6 function in murine lymphocytes, on the other hand, results in increased numbers of B cells, elevated IgE and increased differentiation of Th2 cells associated with atopic dermatitis, highlighting the importance of tight regulation of STAT6 activation for normal immunological function [10]. In support of a role for genetic variation in STAT6 in human allergic disease, many single nucleotide polymorphisms (SNP) of STAT6 are associated with atopic disorders, including atopic dermatitis, eczema herpeticum, food allergies, eosinophilic esophagitis and asthma [11–13]. A GOF effect through increased STAT6 promoter activity has been demonstrated in vitro for the intron 2 SNP rs324011 explaining the high levels of IgE associated with this polymorphism [14].

Recently, a number of case studies have described unrelated and familial atopy reported to have germline missense variants in STAT6 that confer GOF [15–18]. However, analysis of the impact of STAT6 GOF in primary cells from patients remains limited with variable results. Therefore, additional studies identifying and validating STAT6 variants resulting in human atopy is important to further understand STAT6 GOF as a monogenic form of atopic disease and will expand our knowledge of pathogenesis, diversity of phenotypes and potential therapeutic targets.

## Methods

### Ethical Statement

All participants provided written informed consent. The NIHR BioResource Rare Diseases project was approved by the national institutional review board (13/EE/0325). Informed consent for obtaining blood and skin biopsy samples for functional assays were taken in accordance with the Declaration of Helsinki and with approvals from local ethics committees with ethical approval (reference numbers 04/Q0501/119 and 06/Q0508/16).

### Western Blot Quantification of STAT6 and pSTAT6 in PBMC

Peripheral blood mononuclear cells (PBMC) from healthy controls and patient were stimulated with IL-4 50-100 ng/ml or unstimulated, were washed and lysed with radioimmunoprecipitation assay (RIPA) buffer (Sigma-Aldrich) containing protease inhibitor cocktail (Roche) for 30 min on ice. Where ruxolitinib was added, this was for 30 min prior to IL-4 stimulation at 10,000U/ml. Supernatants were collected following centrifugation at 12,000 rpm for 20 min. Proteins were resolved on precast 4–20% Mini-PROTEAN TGX Stain-Free Gels (Bio-Rad) and transferred onto nitrocellulose membranes of Trans-Blot Turbo Transfer Packs (Bio-Rad). Following blocking in TBS-Tween (0.1%, TBST) buffer containing 5% BSA for 1 h at room temperature, membranes were incubated with the antibodies specific for STAT6, pSTAT6 and GAPDH (D3H4 at 1:1000, D8S9Y at 1:1000, and B8G3 at 1:5000 respectively, all Cell Signalling Technology), overnight at 4 °C. Membranes were incubated with anti-mouse HRP-conjugated secondary antibody for 1 h at room temperature. Finally, membranes were incubated in SuperSignal West Pico PLUS Chemiluminescent Substrate (Thermo Scientific) and imaged with the ChemiDoc Imaging System (Bio-Rad). Images were analysed using Image Lab software version 6.1.0, with ratios derived from automated densitometry relative to GAPDH.

### Quantification of Th Subsets by Flow Cytometry

Peripheral blood mononuclear cells were isolated from freshly collected blood by Ficoll-Paque (GE Healthcare) gradient. Cells were surface stained with anti-human anti-CD4 (clone SK3, BD Biosciences), anti-CD45RA (clone HI100, eBiosciences), anti-CXCR3 (clone G025H7, Biolegend) and anti-CCR6 (clone 11A9, BD Biosciences), and washed twice with FACS buffer. Data were analysed within a CD4 + CD45RA- gate and results are shown as percentage of cells.

### Quantification of Total and Phosphorylated STAT-6 by Phosflow

1 × 10^6^ cells resuspended in media were stimulated with IL-4 (10 ng/ml) for 10 min at 37 °C, then fixed in pre-warmed BD Phosflow™ Fix I Buffer (BD Biosciences 557,870) at 37 °C for 10 min. Cells were then permeabilized in BD Phosflow™ Perm Buffer III (BD Biosciences) on ice for 30 min. Following two washes with Wash Buffer, cells were incubated for 1 h at room temperature (RT) with respective antibodies: anti-STAT6 (Clone 23 560,001) and anti-Stat6 (pY641) Alexa Fluor 647 (Clone 18 612,601) (BD Biosciences), and anti-human CD3 (Biolegend 344,804). Stained cells were then washed and resuspended in FACS buffer.

### Immunofluorescence Staining of Fibroblasts with Confocal Microscopy Analysis

7 × 10^4^ cells were seeded in 1 ml of media in a 12 well plate. 24 h later, the cells were stimulated with 50 ng/ml IL-4 for 10 min at 37 °C. The cells were fixed with 4% formaldehyde (Thermo Scientific) for 15 min at RT and washed three times with 1 ml PBS. For phosphoprotein detection, the cells were permeabilized with ice-cold methanol for 10 min at -20 °C. After two further washes with PBS, the cells were blocked in PBS containing Triton (0.3%), BSA (2%) and FCS (2%) for 1 h at RT. Following an overnight incubation with primary antibodies (anti-pSTAT6, clone 18 cs56554, anti-STAT6, rabbit clone 23 cs9362, anti-STAT6, D-1 mouse sc-374021, and anti-tubulin, 9F3 cs2128, at 1:500 dilution, BD Biosciences) in PBS-Triton (0.3%) supplemented with 1% BSA, the cells were washed once with PBS-Triton (0.3%) and twice with PBS for 5 min each wash. Incubation with the respective secondary antibodies (goat anti-rabbit IgG (H + L) cross-absorbed Alexa Fluor™, 568 (A11036) and anti-mouse IgG (H + L) cross-absorbed Alexa Fluor™, 488 (A11001) at RT for 1 h. Following three washes with PBS, coverslips were mounted on VECTASHIELD (Vector Laboratories) containing DAPI. The images were acquired on confocal microscope (Nikon Eclipse Ti), images were analysed using Fiji ImageJ2 Version 2.3.0/1.53q.

### Determination of mRNA Levels by Reverse Transcription Real Time-Quantitative Polymerase Chain Reaction (RT-qPCR) in CD4 T Cells

CD4 T cells were left untreated or were stimulated with 100 ng/ml IL-4 for 12 h. Total RNA was extracted using Monarch Total RNA Miniprep Kit (New England Biolabs). RNA was reverse-transcribed using LunaScript-RT SuperMix Kit (New England Biolabs) and used to quantify the expression levels of BCL-6, EPAS-1, XBP-1, and GATA-3 in triplicates. For quantification of cDNA, qPCR was performed using CFX96 Touch Real-Time PCR Detection System (Biorad) with Taqman probes (ThermoFisher Scientific) labelled with FAM (Hs00153368_m1 for BCl-6, Hs01026149_m1 for EPAS-1, Hs00231936_m1 for XBP-1, Hs00231122_m1 for GATA-3, Hs02786624_m1 for GAPDH, and Hs02800695_m1 for HPRT1). Fold changes were calculated using the ΔΔCt method and results were normalized to the levels of HPRT1 and GAPDH [19].

### Statistical Analysis

Statistical analysis was performed using GraphPad Prism Version 9.2. Data were compared using the Mann–Whitney t-test. Results were expressed as mean. *P*-values < 0.05 were considered significant.

### Other Methods

Cell isolation, cytokine production, construction of STAT6 lentiviral transfer vector, production of stable cell lines, meso scale discovery (MSD) electrochemiluminescence immunoassay and genetic sequencing are described in Supplemental Methods section.

## Results

### Clinical Presentation

The proband in this study presented in his first month of life with severe widespread eczema (Fig. 1B). He was referred for immunological assessment at 1 year old, with suspected hyper-IgE syndrome following recurrent episodes of bacterial pneumonia at age 6 months and 1 year of age and IgE > 5000 kU/L (over detection limit at the time). Thereafter, he had no further episodes of pneumonia. He developed multiple food allergies to cow’s milk, soya, and most tree nuts in infancy and has had 4 episodes of anaphylaxis requiring resuscitation, secondary to food allergy to date. He developed further food allergies during later childhood including to chicken, sesame, cod fish, salmon fish and tuna. He was diagnosed with asthma at age 4 years and had frequent exacerbations throughout childhood, requiring multiple hospital attendances for treatment with nebulised and intravenous salbutamol. He has seasonal allergic rhinoconjunctivitis with documented sensitisation to grass and tree pollen. He was diagnosed with eosinophilic oesophagitis at 36 years of age after presenting with dysphagia and treated with oral budesonide. Currently at 42 years old, his eczema and asthma symptoms are stable. He is treated with inhaled steroids and topical emollients and requires occasional topical steroid use. He has not previously received biological therapies for atopic disease. Immunological investigations in adulthood revealed eosinophilia (16–20% of total white cell count, 1.2–1.44 × 10^9^/L, normal range 0–0.5 × 10^9^/L). His other immunological parameters including IgG, IgA and IgM and full blood count and lymphocyte counts are within the normal range for age. T cell phenotyping found normal populations of naïve and memory T-cells (Supplemental Fig. 1A) and cells expressing markers for Th2, Th1 and Th17 subsets (Fig. 1C–F and Supplemental Fig. 1B–E). In view of the severe history of atopy, we further analysed intracellular cytokines typically associated with a Th2 immunological phenotype. Although the percentage of CD4 cells expressing IL-4 did not differ between the patient and a group of healthy controls (Fig. 1G), a higher mean fluorescent intensity (MFI) of IL-4 in the patient’s CD4 T cells was observed (Fig. 1H). Similar findings were seen in CD8 T cells with a similar proportion of CD8 T cells producing IL-4 compared with control (Fig. 1I) but at a higher MFI (Fig. 1J). In keeping with enhanced IL-4 production, possibly by multiple cell types, a higher level of serum IL-4 was seen in patient serum compared to a group of healthy control sera (Fig. 1K). The percentage of CD4 T cells expressing other Th2 cytokines IL-13 and IL-5 were comparable between the patient and healthy control groups as were the MFI of IL-13 and IL-5 in CD4 T cells and the concentration of IL-13 in serum (Supplemental Fig. 2A–F). The proband’s family is of white, European origin and his parents are non-consanguineous (Fig. 1B).

The proband’s mother presented in early childhood with severe eczema and other typical atopic disease manifestations, including eosinophilia (20% of total white cell count, 1.74 × 10^9^/L, normal range 0–0.5 × 10^9^/L), elevated IgE fluctuating between 7000 to 14,000 IU/ml, multiple food allergies and asthma. Asthma and eczema symptoms ameliorated throughout adolescence, and she continues to have mild symptoms, which are currently stable on inhaled or topical steroids without the requirement for biological therapies. She developed eosinophilic oesophagitis responsive to topical steroid therapy at age 26 years. She was diagnosed with follicular lymphoma (FL) at age 49 years following presentation with bilateral axillary lymphadenopathy, which was treated with standard chemotherapy protocols. She subsequently relapsed with transformed FL (diffuse large B cell lymphoma, DLBCL) at age 60 years. The proband’s younger brother also had severe atopic disease manifestations from early childhood including eczema, asthma with frequent exacerbations, and multiple food allergies including to cow’s milk, nuts, soya, and shellfish. He died of anaphylaxis at age 20 years following ingestion of the non-steroidal anti-inflammatory drug ibuprofen.

Whole genome sequencing was performed for the proband as part of the NIHR Bioresource Rare Diseases study. Pathogenic variants in genes associated with atopic disease and inborn errors of immunity were interrogated but none found [20]. Variants previously associated with disease in the clinical mutation database ClinVar (https://www.ncbi.nlm.nih.gov/clinvar/) were also not found in the patient. Heterozygous *STAT6* c.1255G > C p.D419H (NM_001178078) was found in the proband sequence. Sanger sequencing of the proband, mother and brother confirmed segregation of the *STAT6* mutation in affected family members (Fig. 1B). *In-silico* pathogenicity predictions: SIFT, PolyPhen2 and CADD (Combined Annotation Dependent Depletion) scores predicted the missense mutation to be damaging to STAT6 (‘deleterious’ (0), ‘probably damaging’ (1), CADD score 29.5) [21–23]. The mutation was not found in healthy control databases ExAC, or gnomAD [24, 25].

### STAT6 D419H Confers Gain of Function in HEK293T Cell Lines

**Fig. 2:**
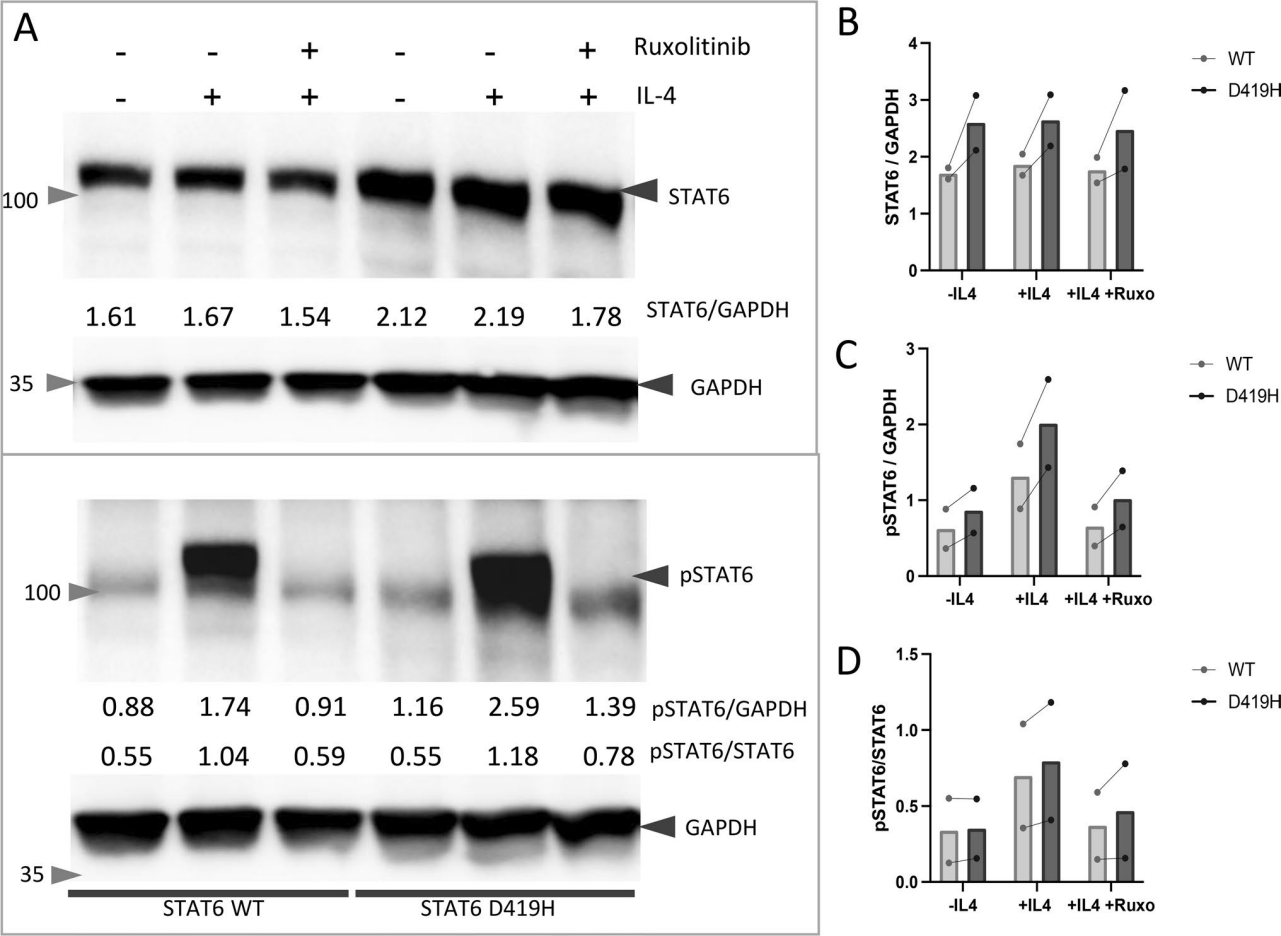
D419H variant leads to increased levels of STAT6 and pSTAT6 in Human embryonic kidney (HEK) 293 T cells.** A**: Western blot with densitometry measurements showing increased STAT6 and pSTAT6 in D419H in HEK 293 T cells, relative to GAPDH housekeeping gene. Image representative of 2 independent experiments. **B, C**: Bar charts show mean densitometry of STAT6 and pSTAT6. **D**: Bar chart show pSTAT6/STAT6 ratio. Data are from 2 independent experiments. Each data point is the mean of 3 technical repeats, with protein levels normalised to GAPDH housekeeping gene. Ruxo- Ruxolitinib

In order to test whether the STAT6 D419H variant supported normal expression and function of STAT6 protein, the human embryonic kidney (HEK) 293 T cell line was stably transduced with lentiviral vectors expressing wild type (WT) and D419H. HEK293T cells were chosen as they lack endogenous STAT6 but express the other proteins required for a functional IL-4/JAK pathway [26]. Green fluorescent protein (GFP) was used as a surrogate marker of transduction and resulting expression of STAT6. Similarly high levels of transduction were seen in WT and D419H cell lines (Supplemental Fig. 3), but levels of total STAT6 were consistently higher in D419H than WT cell lines when measured by western blot in 2 independent experiments (Fig. 2A–B). Low levels of pSTAT6 were seen at baseline in both control and D419H cell lines but pSTAT6 was upregulated to higher levels in D419H than control cells after IL-4 stimulation (Fig. 2A, C). Ruxolitinib, a JAK1 and 2 inhibitor, reduced pSTAT6 levels to baseline in WT cells, whereas this effect was partial in D419H cells (Fig. 2A). The ratio of pSTAT6:STAT6 were similar in D419H compared to WT suggesting that higher levels of pSTAT6 are predominantly due to higher expression of total STAT6 protein (Fig. 2D).

### STAT6 D419H Confers Gain of Function in Primary Non-haematopoietic and Haematopoietic Cells

**Fig. 3:**
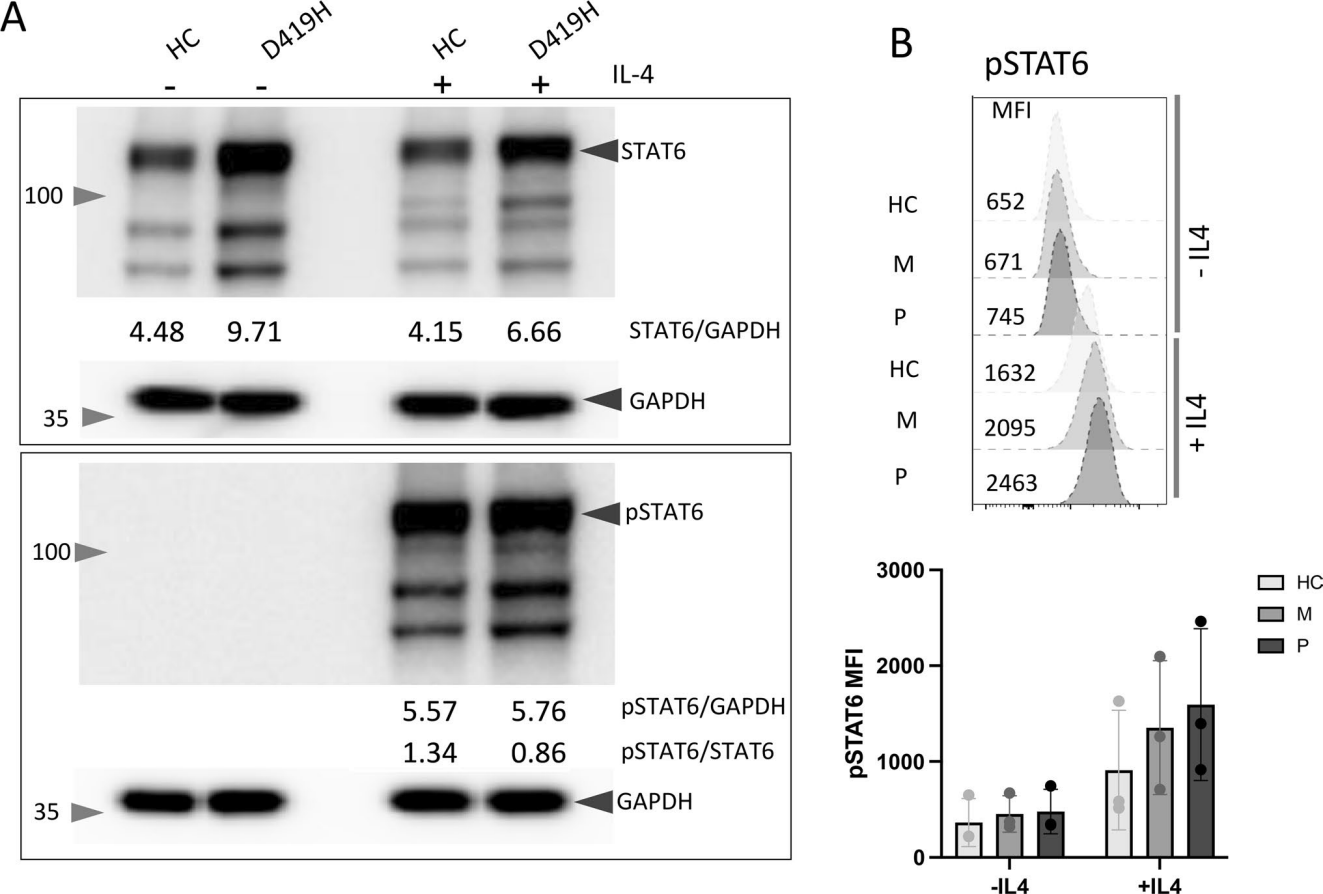
D419H variant leads to increased levels of STAT6 in patient fibroblast cells compared to healthy control (HC).** A**: Western blot with densitometry measurements showing increased STAT6 pre- and post-IL-4 stimulation in D419H fibroblasts, relative to GAPDH housekeeping gene. pSTAT6 was only detected post-IL-4 stimulation in HC and D419H. Image representative of 3 independent experiments. **B**: Flow cytometric analysis showing increased STAT6 and pSTAT6 levels pre- and post IL4 stimulation of D419H fibroblasts from 3 independent experiments. HC – healthy control, M – proband’s mother, P – proband

**Fig. 4:**
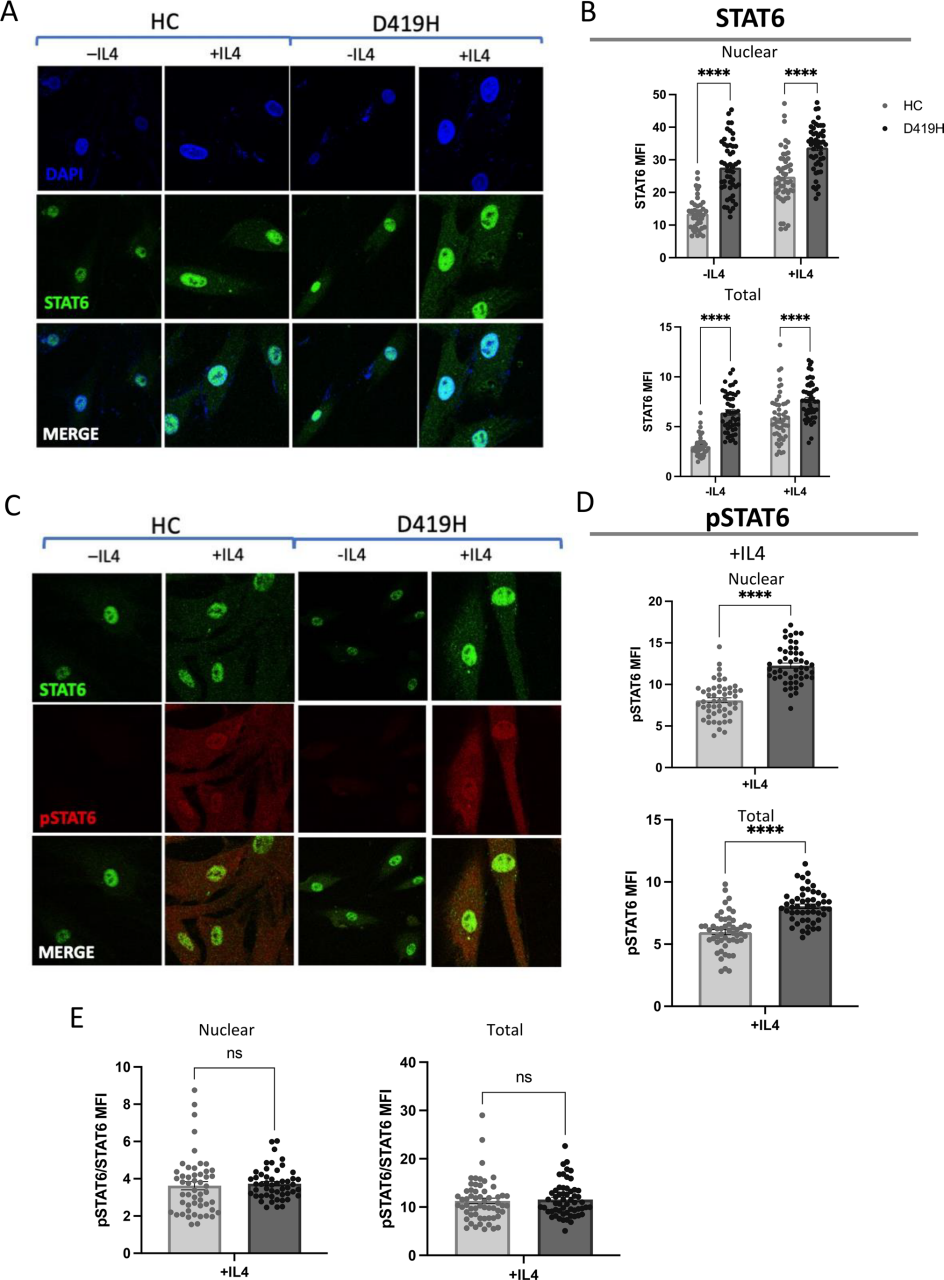
D419H variant leads to increased levels of STAT6 and pSTAT6 in patient fibroblast cells compared to healthy control (HC) with increased nuclear localisation.** A**: Confocal images of immunofluorescence of STAT6 with nuclear localization in D419H proband fibroblasts in comparison to HC, pre- and post- IL-4 stimulation. **B**: Bar charts show mean fluorescent intensity of nuclear and total STAT6 and is representative of 2 independent experiments, with each point representing one cell, 50 cells analysed per condition. **C**: Confocal images of immunofluorescence of pSTAT6 with nuclear localization in D419H proband fibroblasts in comparison to HC, pre- and post- IL-4 stimulation. **D**: Bar charts show mean fluorescent intensity of nuclear and total pSTAT6 following IL-4 stimulation. This is representative of 2 independent experiments, with each point representing one cell, 50 cells analysed per condition. **E**: Bar charts showing ratio of pSTAT6:STAT6 post- IL-4 stimulation in nucleus and in the whole cell. **** *p*-value < 0.0001, ns – not statistically significant

To test whether STAT6 overactivity in HEK293T cells was an artefact of our overexpression cell line model, we generated skin fibroblast cultures from the proband and a healthy control. As seen with HEK239T cells, levels of total STAT6 were higher in D419H proband-derived fibroblasts than healthy control (HC) skin fibroblasts as determined by western blot, both at baseline and following IL-4 stimulation (Fig. 3A and Supplemental Fig. 4). Following IL-4 stimulation, high levels of pSTAT6 were observed in both D419H and HC fibroblasts (Fig. 3A, B). Confocal analysis of skin fibroblasts demonstrated that the majority of STAT6 was already present in the nucleus prior to stimulation in both HC and D419H fibroblasts (Fig. 4A), consistent with continuous STAT6 nuclear trafficking [27]. Significantly higher levels of STAT6 were present in both the cytoplasm and nucleus in D419H fibroblasts than in HC (Fig. 4A, B). As with western blotting, pSTAT6 was only observed after IL-4 stimulation in HC and D419H fibroblasts (Fig. 4C, D) and appeared in both the cytoplasm and the nucleus. Both total cellular and nuclear levels of pSTAT6 were significantly higher in D419H fibroblasts than HC following IL-4 stimulation (Fig. 4D). The ratio of pSTAT6:STAT6 was similar between D419H and control fibroblasts (Fig. 4E).

**Fig. 5:**
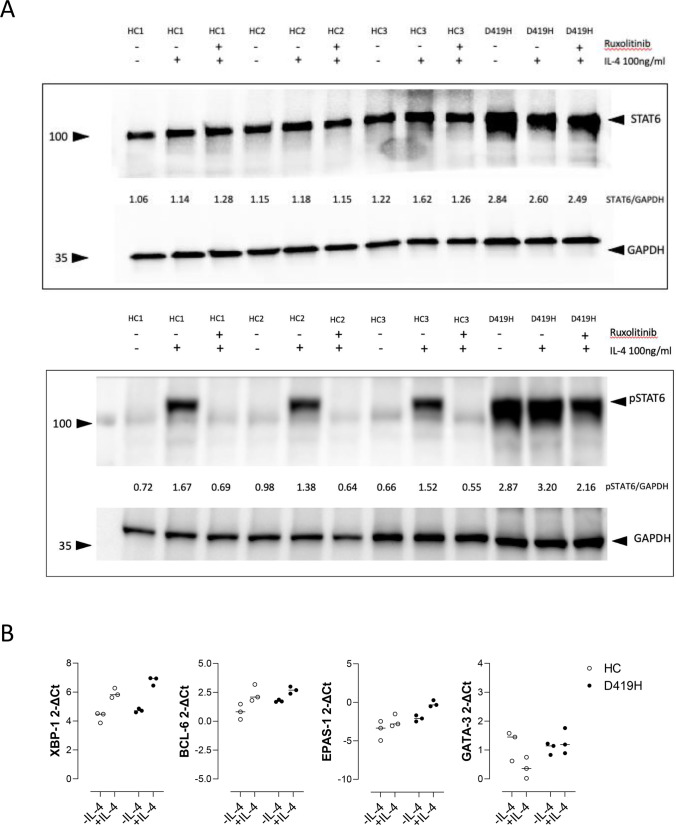
D419H variant leads to increased levels of STAT6 and pSTAT6 in patient PBMC compared to 3 healthy controls (HC).** A**: Western blot with densitometry measurements showing increased STAT6 across IL-4 concentration and with ruxolitinib, in D419H PBMC, and is representative of 2 experiments. **B**: RT-qPCR 2-∆CT levels of XBP-1, BCL-6, EPAS1 and GATA3 mRNA transcript in HC and patient D419H T cells. Lower 2-∆CT values indicate higher gene expression level

To study the impact of D419H in immune cells, we obtained peripheral blood from the proband. Measured by western blot, baseline levels of STAT6 in peripheral blood mononuclear cells (PBMC) were higher in D419H than HC samples. IL-4 stimulation had minimal impact on STAT6 levels but induced pSTAT6 in both HC and D419H PMBC, with higher levels of pSTAT6 seen in D419H cells (Fig. 5A). The ratio of pSTAT6:STAT6 was similar between HC and D419H cells supporting our previous observation in transduced HEK293T cells and fibroblasts (Figs. 2A and 4E). *In-vitro* treatment with ruxolitinib abolished pSTAT6 in HC PBMC and reduced pSTAT6 in D419H, but did not have a noticeable effect in reducing total STAT6 levels within the timeframe of the experiment (Fig. 5A).

### Upregulated Transcriptional Activity of STAT6 Mediated Genes

To test whether increased STAT6 signalling in D419H resulted in altered gene transcription, we analysed the relative expression levels of genes known to be regulated, or partially regulated by STAT6 [6, 7, 28–30] in isolated CD4 T cells. Following IL-4 stimulation, mRNA transcripts for *XBP1*, *EPAS1* and *BCL6* were upregulated in D419H CD4 + T cells when compared to WT control (Fig. 5B). Baseline mRNA levels were also higher in D419H CD4 T-cells compared with HC for *BCL6* and *EPAS1*, suggesting induction of STAT6 target mRNA production even prior to IL-4 stimulation. GATA3 mRNA values were not induced by IL-4 stimulation in HC or D419H cells, in keeping with the known STAT6-independent regulation of GATA3 [31].

## Discussion

STAT6 is an intracellular transcription factor known to be important for regulation of genes critical for Th2 responses that are the hallmark of atopy, including Th2 cell differentiation to promote B cell survival, M2 macrophage polarisation, and class switching to IgE [5, 6]. Here, we report a kindred with STAT6 gain of function disease with severe early onset atopy and follicular lymphoma, associated with a novel germline heterozygous mutation *STAT6* c.1255G > C p.D419H located in the STAT6 DNA binding domain that co-segregated with clinical disease.

Using a combination of constitutive overexpression in the HEK293T cell line and primary patient skin fibroblasts and PBMC, we demonstrate that the D419H mutation confers gain of STAT6 function. Our findings build on reports of patients with different de novo germline STAT6 mutations, associated a similar atopic phenotype as seen in our patient including treatment resistant atopic dermatitis, hyper-eosinophilia, eosinophilic oesophagitis and multiple food allergies [15–18, 32]. Since initial submission of this manuscript four other publications have detailed additional patients with STAT6 GOF disease, including a consortium study that includes the clinical information of the kindred we investigate here [15–18]. Our observation of relapsed lymphoma in the mother is not shared amongst the 20 other cases reported to date but is significant given the association of somatic STAT6 mutations associated with lymphoma [33, 34]. In this study, we provide functional validation, that the D419H STAT6 mutation confers gain of function activity not only in a HEK293T cell line constitutive overexpression models but also in patient haematopoietic and non-haematopoietic primary cells, confirming STAT6 GOF as a dominantly inherited monogenic cause of severe atopy.

In our study, we observed elevated levels of STAT6 and pSTAT6 in D419H cells associated with increased transcription of STAT6-induced genes. The mechanism by which D419H causes GOF is currently unclear. The most straightforward explanation is that the location of the mutation in the DNA-binding domain results in increased or prolonged interaction with STAT6 consensus binding sites as previously suggested for other DNA-binding domain mutations including other mutations at the D419 residue [33]. However, unlike STAT1, STAT6 is not described to regulate its own expression and therefore the mechanism by which the D419H mutation results in elevated total STAT6 levels is not clear [35]. STAT6-mediated gene transcription depends on the presence of other transcriptional cofactors to enhance or repress expression of a large number of downstream genes [5, 36]. It is therefore possible that mutations modify the interaction of STAT6 with binding partners in the transcription complex resulting in altered expression of a gene that in turn regulates STAT6 expression. Alternatively, mutations at the D419 site may alter the specificity of STAT6 DNA binding, as has been described for a neighbouring H415N mutation, with impact on the range of downstream genes, enhanced or repressed, potentially including genes not typically regulated by STAT6 [4]. Here we tested a limited range of STAT6 downstream genes to demonstrate GOF and broader transcriptional studies in specific cell types would be required to better understand the broader impact of STAT6 GOF on immunity. Somewhat surprisingly, we did not find any difference in the frequency of Th1, Th2 or Th17 cells in peripheral blood from the patient, despite the known role of STAT6 in Th2 differentiation and the description of increased Th2 T-cells in some other STAT6 GOF patients [15, 17, 18]. We did observe increased levels of IL-4 cytokine production but not of other Th2 cytokines, IL-5 and IL-13, suggesting that the impact of STAT6 GOF is complex and may be variable between individual mutations or patients.

Based on our findings, it is unlikely that STAT6 GOF is caused by enhanced STAT6 phosphorylation as the ratios of pSTAT6/STAT6 were similar between D419H and healthy control cells, suggesting that the increase in pSTAT6 is predominantly caused by an overall increase in STAT6 levels. While phosphorylation of STAT6 is a well described mechanism for its activation during IL-4 signalling and increased levels of pSTAT6 would be expected to enhance signalling, unphosphorylated STAT6 has also been shown to be constitutively present in the nucleus, as we also observed, and regulate downstream gene activation [37, 38]. The relative contribution of pSTAT6 and unphosphorylated STAT6 to GOF is important to determine as it has potential implications for the use of JAK inhibitors in the management of patients with STAT6 GOF disease. In our experiments, the JAK1/2 inhibitor, ruxolitinib, substantially reduced pSTAT6 in control and D419H HEK293T cells and PBMC, but the impact of ruxolitinib on total STAT6 levels and gene expression were not studied and would require a longer experimental time-course.

An important clinical finding in our kindred, not described in other kindreds, was the co-occurrence of follicular lymphoma (FL) with atopy in STAT6 GOF disease. This is not entirely surprising given the well described association of somatic STAT6 GOF mutations with various lymphomas, including FL [33, 34]. In a focused study of FL, 11% of a cohort of 114 follicular lymphomas had somatic STAT6 GOF mutations with most mutations occurring at the DNA binding interface of STAT6 and the most frequent somatic mutations occurring at amino acid 419, which was described as a hotspot for genetic mutation (D419G, D419A, and D419H) [33]. Lymphoma cell lines bearing these DNA-binding domain mutations permitted constitutive expression of IL-4-related genes that did not depend on STAT6 tyrosine phosphorylation at the Y641 site, suggesting a mechanism by which STAT6 GOF may promote lymphomagenesis.

Our study describes a kindred with a single DNA-binding domain STAT6 GOF mutation resulting in severe atopy associated with FL. A number of important questions remain to be answered in future studies. Description of additional cases will extend our understanding of the clinical phenotype associated with germline STAT6 GOF mutations and enable comparison of disease mechanism between different mutations. Better understanding of the molecular mechanisms of disease will be important to inform targeted therapy, which may have broader impact for other forms of allergic disease beyond this specific monogenic cause.

## Supplementary Information

Below is the link to the electronic supplementary material.

Supplementary file1 (DOCX 2655 KB)

Supplementary file2 (DOCX 22 KB)

## Data Availability

The datasets generated during and/or analysed during the current study are available from the corresponding author on reasonable request.

## Abbreviations

GOF: Gain of function
HEK: Human Embryonic Kidney
IFNγ: Interferon gamma
IL-4: Interleukin-4
IL-5: Interleukin-5
IL-13: Interleukin-13
IL-17: Interleukin-17
JAK: Janus Kinase
MFI: Mean fluorescence intensity
PBMC: Peripheral blood mononuclear cells
PMA: Phorbol 12-myristate 13-acetate
pSTAT: Phosphorylated Signal Transducer and Activator of Transcription
RT: Room temperature
SNP: Single nucleotide polymorphism
STAT: Signal Transducer and Activator of Transcription
Th2: T-helper -2
WT: Wild type
