# Supplementary material for: Autosomal Dominant STAT6 Gain of Function Causes Severe Atopy Associated with Lymphoma
Source: J Clin Immunol. 2023 Jun 14;43(7):1611–22. doi: 10.1007/s10875-023-01530-7 (PMC10499697; doi:10.1007/s10875-023-01530-7)
Supplement: Supplementary file 1 — Supplementary file1 (DOCX 2655 KB) [file 10875_2023_1530_MOESM1_ESM.docx]

**Supplemental Figure 1.** **T cell phenotyping in proband and healthy controls.** Flow cytometric analysis of naïve/memory T cells of healthy control and proband cells from peripheral blood **(A)**, CD4 and CD8 T cells producing Th1/Th2/Th17 cytokines of healthy control and proband **(B)**. **C**: Frequency of CD4 T cells expressing IL-17 **(C)**, expressing IFNγ **(D)** and frequency of CD8 T cells expressing IFNγ **(E)** as compared with a cohort of healthy controls. Each dot represents one individual.

**Supplemental Figure 2**: **A**: Representative flow cytometry analysis of IL-13 and IL-5 producing CD4+ T cells in D419H and healthy controls (HC). Frequency of CD4+ T cells producing IL-13 **(B)**; IL-13 MFI within CD4+IL4+ cells **(C)**; Frequency of CD4+ T cells producing IL-5 **(D)**; IL-5 MFI within CD4+IL4+ cells **(E)**. Serum IL-13 levels **(F)** as determined by multiplex electrochemiluminescence-based cytokine assay. Each dot represents one individual.

**Supplemental Figure 3:** Gating strategy for GFP HEK293T cells

**Supplemental Figure 4: D419H variant in proband and mother leads to increased levels of STAT6 in fibroblast cells compared to 4 healthy controls (HC).** Western blot with densitometry measurements showing increased STAT6 pre- and post-IL-4 stimulation in D419H fibroblasts, relative to GAPDH housekeeping gene. Image representative of 4 independent experiments.
