# Supplementary material for: Autosomal Dominant STAT6 Gain of Function Causes Severe Atopy Associated with Lymphoma
Source: J Clin Immunol. 2023 Jun 14;43(7):1611–22. doi: 10.1007/s10875-023-01530-7 (PMC10499697; doi:10.1007/s10875-023-01530-7)
Supplement: Supplementary file 2 — Supplementary file2 (DOCX 22 KB) [file 10875_2023_1530_MOESM2_ESM.docx]

**Supplemental methods**

**Cell isolation**

Peripheral blood mononuclear cells (PBMCs) were isolated from freshly collected blood by Ficoll-Paque PLUS (GE Healthcare). CD4 T-cells were subsequently purified by negative selection (purity > 96%, MiltenyiBiotec).

**Cytokine production at the single-cell level**

Cytokine production was assessed at the single cell level after 4h stimulation of PBMCs with phorbol 12-myristate 13-acetate (PMA, Sigma-Aldrich) 50ng/ml plus Ionomycin (Sigma-Aldrich) 500ng/ml in the presence of Brefeldin A (Sigma-Aldrich) 10μg/ml. After stimulation, cells were surface stained with anti-human anti-CD3 (clone UCHT1, BD Biosciences), anti-CD8 (clone SK3, BD Biosciences), anti-CD4 (clone SK3), anti-CD45RA (clone HI100, eBiosciences) and anti-CD27 (Clone L128, BD Biosciences) followed by fixation with BD Cytofix/Cytoperm (BD Biosciences) and permeabilized with Perm/wash Buffer (BD Biosciences). Cells were intracellularly stained with anti-human anti-IL-17 (clone SCPL1362, BD Biosciences), anti-IFN-γ (Clone B27, BD Biosciences), anti-IL4 (clone 8D4-8) and anti-IL2 (clone MQ1-17H12) for 30 min or with anti-IL-5 (clone TRFK5, Biolegend) and IL-13 (clone JES10-5A2, BD Biosciences) for 18 hours.. Results are shown as frequency of CD4 or CD8 T cells producing a given cytokine, or the MFI of a given cytokine within a CD4+ or CD8+ T cell gate and compared with a group of healthy controls (unpublished data). -17H12

**Flow cytometry**

A minimum of 150,000 events were acquired on a BD LSRFortessa (BD Biosciences) and data was analyzed using FlowJo software (TreeStar). After lymphocyte gate definition, doublets were excluded, and cells analyzed within the mentioned gates.

**Construction of STAT6 lentiviral transfer vector and production of stable cell lines**

To construct a lentiviral transfer vector, the sequence encoding STAT6 WT was amplified with FwSTAT6_XhoI (5’-gcgctcgagcatgtctctgtggggtctggtc-3’) and RevSTAT6_BamHI (5’-GCGggatccCCAACTGGGGTTGGCCCTTAG-3’) primers incorporating *Xho*I and *BamH*I restriction sites for cloning. The PCR product was digested with *Xho*I and *BamH*I restriction enzymes and cloned into pLV T2A GFP, similarly restricted. The D419H mutation was introduced by recombination of two PCR products in competent JM109 cells (Promega). The first was amplified with forward primer Fw STAT6 D>H Mut (5’-GTCATCGTCCATGGCAACCAACACAACAATGCCAAAGC-3’) encoding the mutated sequence and a reverse primer RevLenti2AGFP (5′- GAAGGCGATGCGCTGCGAATCGGGAGC -3′) binding to the vector backbone. The second was amplified using a reverse primer Rev STAT6 D>H Mut (5’-CAGGATAGTGGCTTTGGCATTGTTGTGTTGGTTGCCATG-3’) encoding the mutated sequence and a forward primer FwLenti2AGFP (5’- GCTCCCGATTCGCAGCGCATCGCCTTC -3’) binding to the vector backbone. As a result, the lentiviral vector co-expressed STAT6 WT (or STAT6 D419H) and the green fluorescent protein (GFP) under the control of PGK promoter *via* T2A (Thosea asigna virus 2A) self-cleaving peptide at equimolar amounts.

293T cells (ThermoFisher) were transfected with 3 μg pMDG2 (VSV-G envelope), 3 μg pCMVRd8.74 (Gag/pol packaging) and 5 μg pLV transfer vector in 200μL OptiMEM (serum-free medium) containing 10 μL FuGENE HD transfection reagent (Promega). Supernatants containing recombinant lentivirus were collected 48 hrs post transfection. Human embryonic kidney (HEK) 293T cells, lacking endogenous STAT6 protein, were used for lentiviral transduction at multiplicity of infection (MOI) of 1. The cells were sorted based on MFI of GFP protein.

**Cell culture.**

All cell lines were cultured in DMEM (Dulbecco’s Modified Eagle Medium) GlutaMAX (Gibco) supplemented with 10% inactivated foetal calf serum (FCS) and penicillin-streptomycin (Sigma) at 37°C in 5% CO_2._

**Genetic sequencing**

Whole genome sequencing of both proband and his affected mother was performed as part of the NIHR Bioresource Rare Diseases study. The sequencing platform was provided by Illumina on the HiSeq X Ten (Illumina, San Diego, CAL), with standard protocols. Data were processed following standard procedures for single nucleotide variants and indels, and copy number variants were called using Illumina workflow. Variants were annotated using the Variant Effect Predictor tool (Ensembl, EMBL-EBI). Bioinformatic analysis and filtering followed to exclude variants of low coverage, low Illumina sequence quality filters, having a minor allele frequency of <0.001 in the Exome Aggregation Consortium (ExAC) data set, and having a minor allele frequency of <0.001 in the NIHR BioResource Rare Diseases data set [21]. Candidate variants were validated by Sanger sequencing with standard protocols.

**Cytokine assay**

Cytokine levels were measured in the serum using a Meso Scale Discovery multiplex kit (Meso Scale Diagnostics, Rockville, Md), according to the manufacturer’s instructions. The lowest level of detection was IL-4 <0.01 pg/ml and IL-13 <0.03 pg/ml.
